# Conserved Bacterial-Binding Peptides of the Scavenger-Like Human Lymphocyte Receptor CD6 Protect From Mouse Experimental Sepsis

**DOI:** 10.3389/fimmu.2018.00627

**Published:** 2018-04-12

**Authors:** Mario Martínez-Florensa, Cristina Català, María Velasco-de Andrés, Olga Cañadas, Víctor Fraile-Ágreda, Sergi Casadó-Llombart, Noelia Armiger-Borràs, Marta Consuegra-Fernández, Cristina Casals, Francisco Lozano

**Affiliations:** ^1^Immunoreceptors of the Innate and Adaptive System, Institut d’Investigacions Biomèdiques August Pi i Sunyer (IDIBAPS), Barcelona, Spain; ^2^Centro de Investigación Biomédica en Red de Enfermedades Respiratorias (CIBERES), Instituto de Salud Carlos III, Madrid, Spain; ^3^Departmento de Bioquímica y Biología Molecular, Universidad Complutense de Madrid, Madrid, Spain; ^4^Servei d’Immunologia, Centre de Diagnòstic Biomèdic, Hospital Clínic de Barcelona, Barcelona, Spain; ^5^Departament de Biomedicina, Facultat de Medicina, Universitat de Barcelona, Barcelona, Spain

**Keywords:** bacteria, CD6, cecal ligation and puncture, infection, peptide interaction, scavenger receptor, sepsis, Imipenem/Cilastatin

## Abstract

Sepsis is an unmet clinical need constituting one of the most important causes of death worldwide, a fact aggravated by the appearance of multidrug resistant strains due to indiscriminate use of antibiotics. Host innate immune receptors involved in pathogen-associated molecular patterns (PAMPs) recognition represent a source of broad-spectrum therapies alternative or adjunctive to antibiotics. Among the few members of the ancient and highly conserved scavenger receptor cysteine-rich superfamily (SRCR-SF) sharing bacterial-binding properties there is CD6, a lymphocyte-specific surface receptor. Here, we analyze the bacterial-binding properties of three conserved short peptides (11-mer) mapping at extracellular SRCR domains of human CD6 (CD6.PD1, GTVEVRLEASW; CD6.PD2 GRVEMLEHGEW; and CD6.PD3, GQVEVHFRGVW). All peptides show high binding affinity for PAMPs from Gram-negative (lipopolysaccharide; *K*_d_ from 3.5 to 3,000 nM) and Gram-positive (lipoteichoic acid; *K*_d_ from 36 to 680 nM) bacteria. The CD6.PD3 peptide possesses broad bacterial-agglutination properties and improved survival of mice undergoing polymicrobial sepsis in a dose- and time-dependent manner. Accordingly, CD6.PD3 triggers a decrease in serum levels of both pro-inflammatory cytokines and bacterial load. Interestingly, CD6.PD3 shows additive survival effects on septic mice when combined with Imipenem/Cilastatin. These results illustrate the therapeutic potential of peptides retaining the bacterial-binding properties of native CD6.

## Introduction

Sepsis is a common and life-threatening disease worldwide causing organ dysfunction as a consequence of dysregulated host inflammatory response to an infection (mainly bacterial) (1). Its more deadly form is septic shock, in which profound circulatory, cellular, and metabolic abnormalities are associated with a greater risk of mortality, particularly for the elderly and the immunocompromised (2, 3). Despite advances in supportive care and availability of potent broad-spectrum antibiotics, the incidence and severity of sepsis and septic shock have been rising steadily as a result of population aging, invasive medical procedures, chronic disease prevalence, and emergence of multidrug resistant (MDR) bugs (1, 3). Sepsis remains an unmet clinical need of complex pathophysiology, calling for urgent innovative developments on cost-effective biological treatments and/or medical devices, alternative or complementary to antibiotics and supportive care (4).

Adjunctive/alternative therapies to antibiotics include host-directed approaches addressed to potentiate the innate defense mechanisms and/or reverse the immune cell dysfunction associated with sepsis mortality (5). The neutralization of pathogenic microbial factors with endogenous host immune constituents represents one such approach. In this regard, some members of the ancient and conserved scavenger receptor cysteine-rich superfamily (SRCR-SF) interact with pathogen-associated molecular patterns (PAMPs) both from Gram-negative (lipopolysaccharide, LPS) or Gram-positive (lipoteichoic acid, LTA and peptidoglycan, PGN) bacteria (6). PAMPs are constitutive components of bacterial walls, absent from the host, and essential for bacterial survival and pathogenicity (7). PAMPs are crucial for bacterial viability and virulence and have remained conserved through evolution The prototypical member of the SRCR-SF displaying bacterial PAMPs binding properties is deleted in malignant brain tumors-1 (DMBT-1), also known as salivary agglutinin (SAG) or gp340 (8, 9). DMBT-1/SAG is a soluble glycoprotein containing 14 SRCR, one zona pellucida, and two C1r/C1s Uegf Bmp1 domains. The bacterial-binding properties of DMBT-1/SAG have been accurately mapped within its SRCR domains to an 11-mer consensus peptide sequence (DMBT-1/SAG.pbs1, GRVEVLYRGSW) from which a 9-mer motif (VEVLxxxxW) present in 13 out 14 of them was identified (10). Other SRCR-SF members with bacterial-binding properties include the following: Class A macrophage scavenger receptor type I (11), macrophage receptor with collagenous structure (MARCO) (12), soluble protein α (13), CD6 (14), CD163 (15), scavenger receptor class A member 5 (16), and soluble scavenger receptor cysteine-rich group B member with five domains (17, 18). The bacterial-binding regions of these peptides have only been functionally mapped for MARCO (12) and CD163 (15).

CD6 is a lymphocyte surface glycoprotein expressed by all T cells and a subset of B and NK cells (19). Functionally, CD6 is a signal-transducing receptor involved in lymphocyte activation and differentiation upon adhesive contacts with antigen-presenting cells expressing the CD6 ligand—CD166/activated leukocyte cell adhesion molecule (ALCAM) (20)—or other recently reported counterreceptors such as Galectins (21) and CD318 (22). Structurally, CD6 belongs to the SRCR-SF owing to the three tandem SRCR repeats in its ectodomain. Previous work by our group demonstrated that the recombinant soluble human CD6 ectodomain (rshCD6) binds to and agglutinates Gram-positive and Gram-negative bacteria *in vitro*. *In vivo*, rshCD6 infusion protects mice from septic shock induced by mono- and polymicrobial models of peritonitis (14, 23, 24). Binding of rshCD6 to bacterial PAMPs such as LPS, LTA, or PGN takes place with *K*_d_ affinities in the nM range, similar to CD14’s binding affinity to the same PAMPs (25, 26). Moreover, rshCD6 downmodulates the pro-inflammatory cytokine (IL-1β, IL-6, and TNF-α) release triggered by LPS or LTA/PGN (23). In light of this evidence, the bacterial-binding properties of CD6-derived peptide sequences (CD6.PD1, CD6.PD2, and CD6.PD3) homologous to the 11-mer consensus peptide previously reported in DMBT-1/SAG (pbs1) were investigated. Our *in vitro* and *in vivo* results support the therapeutic potential of these peptide sequences, with varying degrees of bacterial agglutination and PAMP binding potential, and demonstrate a protective role in cecal ligation and puncture (CLP)-induced polymicrobial peritonitis (27).

## Materials and Methods

### Production and Purification of Recombinant Proteins and Peptides

Recombinant soluble human CD6 ectodomain and rshCD5 proteins were purified following the reported methods (28) using SURE CHO-M Cell line™ clones (Selexis SUREtechnology Platform™, Geneva, Switzerland) and size-exclusion chromatography protocols developed at PX’Therapeutics (Grenoble, France). Human and bovine seroalbumin (BSA) were purchased from Sigma-Aldrich (St. Louis, MO, USA). Peptides (>80% purity) were manufactured by ProteoGenix (Schiltigheim, France) and stocked at 5 mg/mL with diluted (1:3) acetonitrile.

### Bacterial Agglutination Assays

5 × 10^8^ colony-forming units (CFU)/mL diluted in TTC buffer (50 mM Tris pH 7.5 plus 150 mM NaCl, 0.1% Tween 20, and 1 mM Ca^2+^) were mixed (1:1) with different peptide concentrations (0–200 µg/mL) in 96 U-bottomed well microtiter plates (Biofil) (29). After overnight incubation at 37°C, bacterial agglutination was examined by light microscopy and scored from − (absent) to + + + (maximal).

### Bacterial Strains

Multidrug-resistant *Acinetobacter baumannii* clinical isolate, *Enterobacter cloacae* ATCC 23355, *Escherichia coli* ATCC 25922, *Klebsiella pneumoniae* ATCC 13883, *Listeria monocytogenes* ATCC 19111, *Pseudomonas aeruginosa* ATCC 27853, *Staphylococcus aureus* ATCC 25923, and Methicillin-resistant *S. aureus* (MRSA) clinical isolate were provided by Dr. Jordi Vila (Microbiology Department, Hospital Clinic of Barcelona) and grown in Luria Bertani or agar with 5% sheep blood (Becton Dickinson) at 37°C, except for *L. monocytogenes* that was cultured in Brain Heart infusion broth (Pronadisa).

### Binding Assays

#### Intrinsic Fluorescence Experiments

To explore the ability of different peptides/proteins to bind LTA (Mr = 14,000, from *S. aureus*; Sigma) and rough LPS (Re-LPS, Mr = 2,500, from *Salmonella minnesota* serotype Re 595; Sigma), binding studies were carried out in an AB2 spectrofluorometer with a thermostated cuvette holder (±0.1°C), using 5 mm × 5 mm path length quartz cuvettes as described (30). Re-LPS concentration was assessed by quantification of 2-keto-3-deoxyoctulosonic acid (31). Peptide/protein samples (10 µg/mL) were titrated with different amounts of a stock solution of either LTA or Re-LPS in phosphate buffered saline (PBS) pH 7.2, and the Trp fluorescence emission spectra recorded with excitation at 295 nm. The fluorescence intensity readings were corrected for the dilution caused by peptide/protein addition. Background intensities in peptide/protein-free samples due to LTA or Re-PS were subtracted from each recording. The apparent dissociation constant (*K*_d_) of peptide/protein–ligand complexes were obtained by non-linear least-squares fitting to the Hill equation of the change in peptide fluorescence at 353 nm with the amount of added LTA or Re-LPS (31): Δ*F/ΔF*_max_ = [*L*]*^n^*/([*L*]*^n^* + *K*_d_), where Δ*F* is the change in fluorescence intensity at 353 nm relative to the intensity of free peptide; Δ*F*_max_ is the change in fluorescence intensity at saturating LTA or Re-LPS concentrations; [*L*] is the molar concentration of free ligand; and *n* is the Hill coefficient.

#### Solid Phase Binding Assays

96-well microtiter plates (Nunc, Roskilde, Denmark) were coated overnight at 4°C with 5 µg/mL of purified LPS (from *E. coli* O111:B4, Sigma L2630) or LTA (from *S. aureus*, Sigma L2515) in PBS and then incubated for 2 h at room temperature in blocking solution (20 mM Tris-HCl pH 7.4 plus 0.05% Tween 20 and 1% BSA). Biotin-labeled peptides/proteins (2.5–20 µg/mL) were added and incubated overnight at 4°C in blocking solution. After extensive washing, bound peptides/proteins were detected by the addition of horseradish peroxidase-labeled streptavidin (1:5,000 dilution; DAKO) for 1 h at room temperature. Color was developed by adding 3,3’,5,5’-tetramethylbenzidine liquid substrate (Sigma) and optical density read at 405–620 nm.

### Dynamic Light Scattering (DLS)

The hydrodynamic diameters of peptides (10 µg/mL in PBS) were measured at 25°C in a Zetasizer Nano S from Malvern Instruments (Worcestershire, UK) equipped with a 633 nm HeNe laser, as described (32). Four scans were recorded for each sample, and the samples were analyzed in triplicate.

### *In Vitro* Cell Cultures

Spleens from 6- to 8-week-old C57BL/6 mice (Charles River) were disaggregated by filtering through a cell strainer and, after erythrocyte lysis, the cells were resuspended in RPMI 1640 with l-glutamine (Lonza) plus 10% fetal calf serum (BioWest), 100 U/mL penicillin, 100 µg/mL streptomycin, and 50 µM 2-β-mercaptoethanol (Merck). Cells (2 × 10^5^) were stimulated for 48 h (at 37°C in a humidified atmosphere with 5% CO_2_) in U-bottomed 96-well plates (Biofil) containing LPS (0.5 µg/mL; *E. coli* O111:B4), in the presence or absence of increasing peptides (0.5–20 µg/mL). Culture supernatants were harvested and mouse cytokines measured by ELISA following manufacturer’s instructions (BD Biosciences OptEIA sets).

### CLP Procedure

Animal procedures were approved by the Animal Experimentation Ethical Committee, University of Barcelona. High-grade mortality (≥90% mortality within the first 48–72 h) CLP-induced septic shock was induced in 8- to 10-week-old C57BL/6J male mice (20–25 g; Charles River) as previously reported (24).

For the assessment of bacterial load, blood and spleen samples from CLP-treated mice were collected, homogenized, and diluted aseptically in sterile PBS. Serial dilutions were plated overnight on agar with 5% sheep blood (Becton Dickinson) at 37**°**C. Viable bacterial counts were expressed as CFU/mL (blood) or per mg (spleen).

### Statistical Analysis

Survival assays were analyzed by a log-rank χ^2^ test using GraphPad Prism software. The significance of differences between experimental groups was determined by two-tailed paired *t* test with 95% confidence interval (CI). *P* values were considered significant when *P* < 0.05. Statistical analysis (mean ± SEM) was performed using a two-tailed Mann–Whitney test, with 95% CI.

## Results

### Induction of Bacterial Agglutination by CD6-Derived Peptides

**Figure 1:**
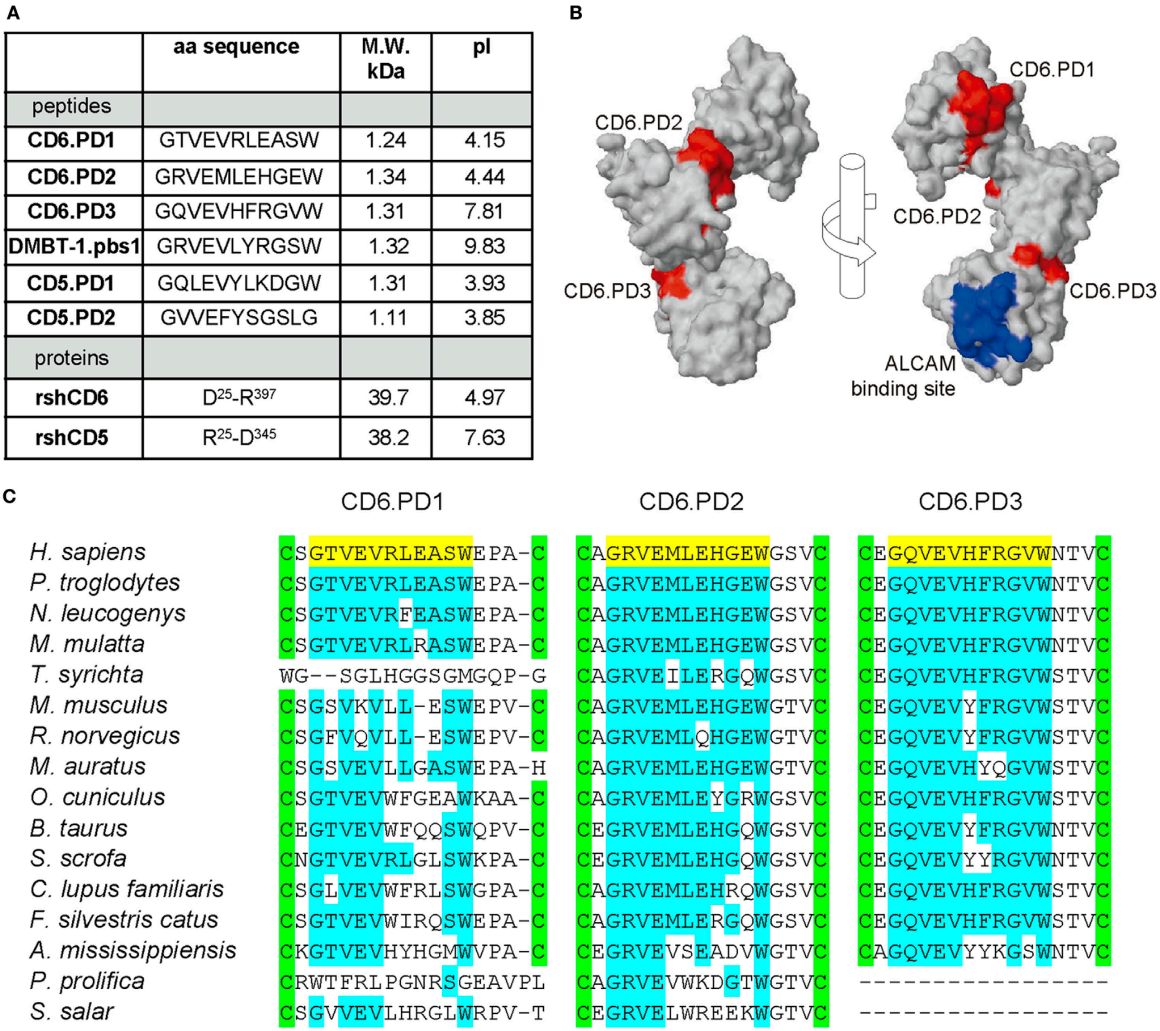
Structural characteristics of the peptides and proteins from the scavenger receptor cysteine-rich superfamily (SRCR-SF) members in the study. **(A)** Amino acid sequence, molecular weight (M.W.) and isoelectric point (pI) of the CD5, CD6, and deleted in malignant brain tumors-1 (DMBT-1) peptides and proteins analyzed in this study. **(B)** Three-dimensional surface representations of the extracellular region of human CD6 (PDB identifier 5a2e, visualized with Jmol) displaying the relative position of the CD6 peptides in the study (colored red) and amino acids involved in binding to the CD6 ligand, CD166/activated leukocyte cell adhesion molecule (ALCAM) (colored blue). **(C)** Alignment of the SRCR domain 1, 2, and 3 amino acid sequences of CD6 from primate (*Homo sapiens*, accession number P30203; *Pan troglodytes*, H2Q3T6; *Nomascus leucogenys*, G1RTX7; *Macaca mulatta*, H9ZFC2; and *Tarsius syrichta*, A0A1U7SL56) rodent (*Mus musculus*, Q61003; *Rattus norvegicus*, Q812A4; and *Mesocricetus auratus*, A0A1U8BJ49), lagomorph (*Oryctolagus cuniculus*, G1T3D3), artiodactyl (*Bos taurus*, F1MU15; *Sus scrofa*, K7GS39), carnivoran (*Canis lupus familiaris*, F1PV91; *Felis silvestris catus*, M3WJ99), crocodilian (*Alligator mississippiensis*, A0A151NXS4), and fish (*Poeciliopsis prolifica*, A0A0S7G288; *Salmo salar*, A0A1S3RVR2) species, where the peptides in study (colored yellow) map. Conserved intradomain cysteine residues and amino acid identities are highlighted in green and blue, respectively.

To investigate the bacterial-binding properties of CD6, intradomain peptide sequences homologous to the consensus 11-mer DMBT-1/SAG.pbs1 peptide sequence (GRVEVLYRGSW) (10) were synthesized. The sequence and physicochemical properties of such CD6-derived peptides mapping at SRCR domains 1–3 (CD6.PD1, CD6.PD2, and CD6.PD3, respectively), as well as of the other peptides and proteins used in this study are compiled in Figure 1A. Structural analyses depicted in Figure 1B showed that all CD6 peptides are accessible at the surface of CD6, with CD6.PD1 (and CD6.PD3) being exposed at opposing sides from that of CD6.PD2. Interestingly, CD6.PD3 mapped at a distant position of the amino acids involved in CD6 binding to CD166/ALCAM (20) (Figure 1B). As illustrated in Figure 1C, the amino acid conservation of the CD6-derived peptides among different animal species was relatively high for CD6.PD2 and CD6.PD3 and lower for CD6.PD1.

**Figure 2:**
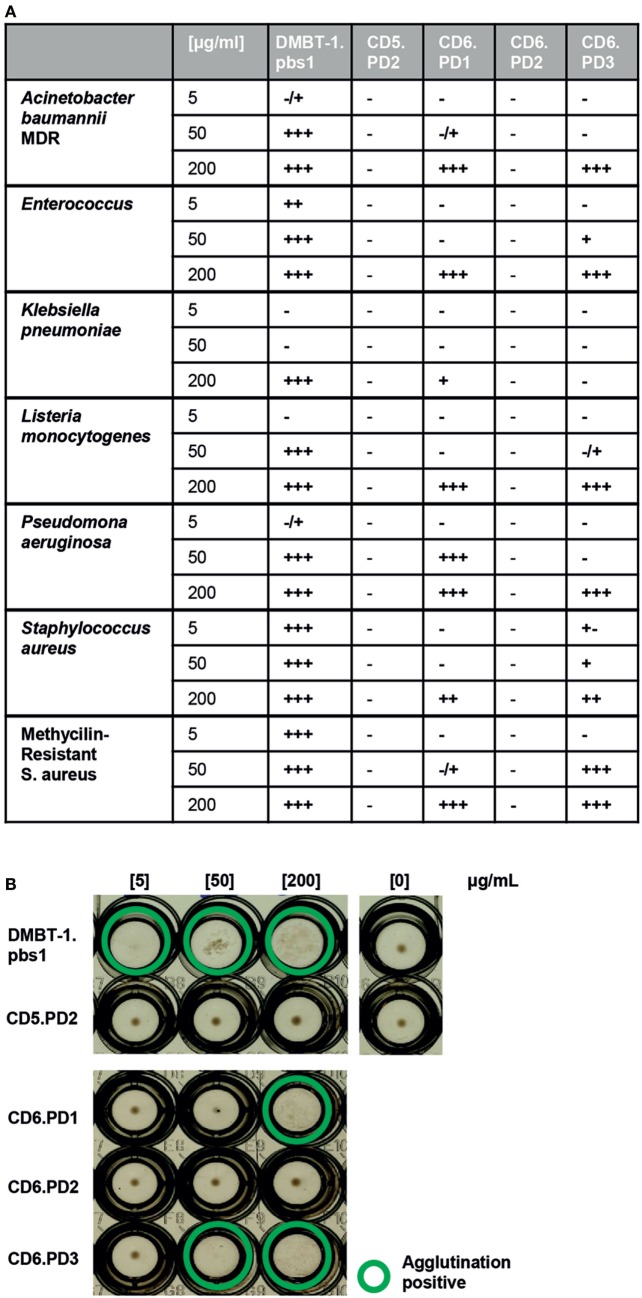
Bacterial agglutination properties of CD6-derived peptides. Increasing concentrations (5, 50, and 200 µg/mL) of the indicated CD6 (PD1, PD2, and PD3), deleted in malignant brain tumors-1 (DMBT-1) (pbs1; C+), and CD5 (PD2; C−)-derived peptides were incubated for 2 h at room temperature in 96-well U-bottomed plates with alive bacterial cell suspensions [75 × 10^6^ colony-forming units (CFU)/mL] in TTC buffer. Bacterial agglutination was scored and consensed by two independent observers as −, ±, +, + +, or +++. **(A)** Summary of the agglutination results obtained with the indicated panel of Gram-negative (multidrug-resistant *Acinetobacter baumannii* clinical isolate; *Enterobacter cloacae* ATCC 23355; *Escherichia coli* ATCC 25922; *Klebsiella pneumoniae* ATCC 13883; *Listeria monocytogenes* ATCC 19111; and *Pseudomonas aeruginosa* ATCC 27853) and Gram-positive [*Staphylococcus aureus* ATCC 25923; methicillin-resistant *S. aureus* (MRSA) clinical isolate] bacterial strains. **(B)** Representative agglutination results obtained for the MRSA clinical isolate.

None of the CD6-derived peptides matched the minimal 9-mer DMBT-1/SAG.pbs1 consensus motif (VEVLxxxxW)—a fact also shared by the CD163p2 (GRIEIKFQRRW) peptide (15)—and function was explored in bacterial agglutination assays. The DMBT-1/SAG.pbs1 peptide was used as positive control (10), and the analogous peptide sequence (CD5.PD2) present in the second SRCR domain of CD5—a highly homologous lymphocyte receptor for which no bacterial binding properties have been reported—used as negative control. As illustrated by Figures 2A,B, dose-dependent agglutination of different Gram-positive and Gram-negative bacterial suspensions (including MDR strains) was observed for CD6.PD1 and CD6.PD3, but not for CD6.PD2. These results indicate that some but not all CD6-derived sequences retain the bacterial agglutination properties of native CD6 (14) and DMBT-1/SAG proteins and of pbs1 (10, 29).

### CD6-Derived Peptides Directly Interact With PAMPs Constitutive of Gram-Negative and Gram-Positive Bacteria With Different Affinities

**Figure 3:**
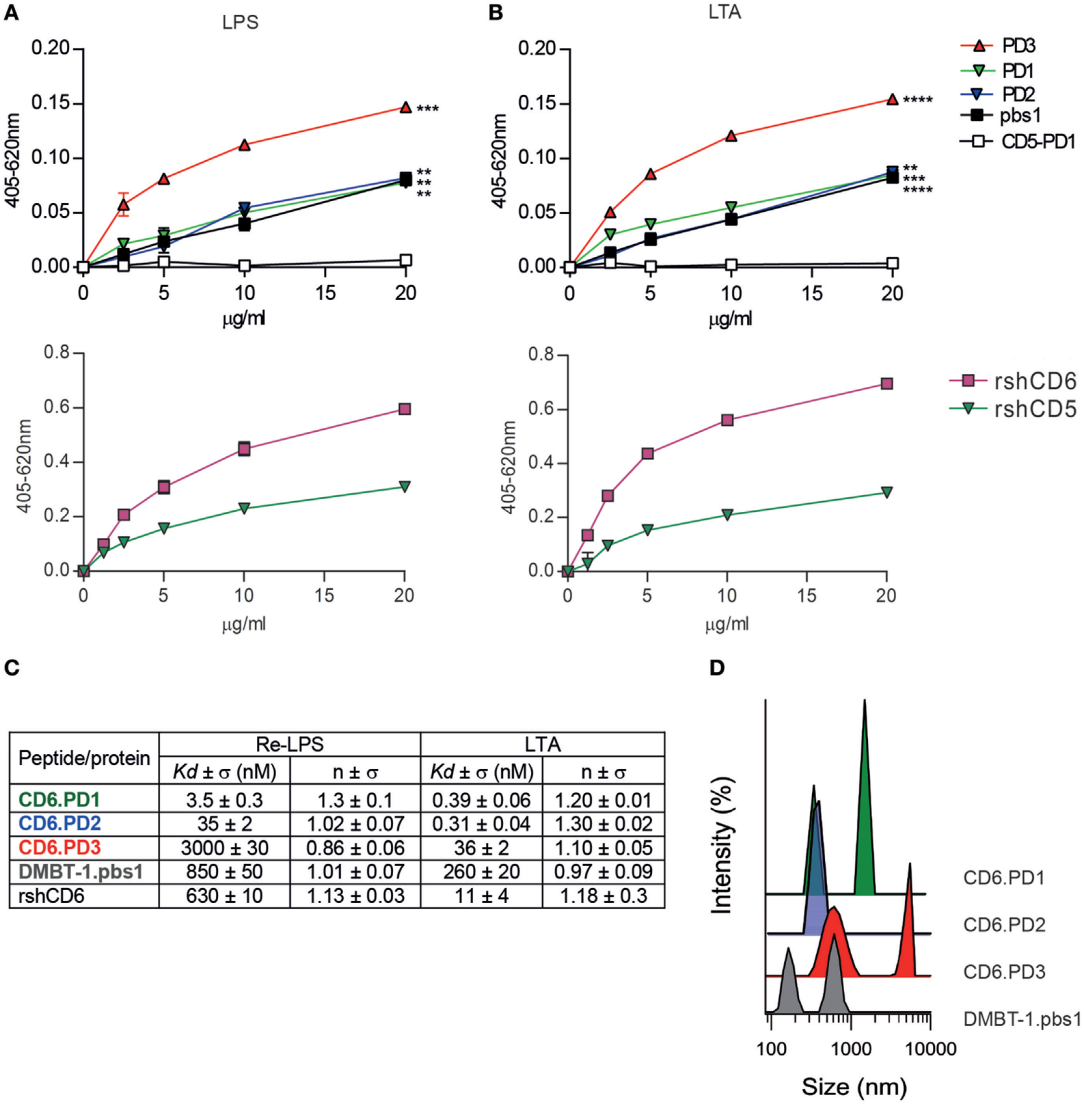
Analysis of the direct binding characteristics of CD6-derived peptides to purified pathogen-associated molecular patterns (PAMPs) from Gram-negative and Gram-positive origin. Increasing concentrations (5–20 µg/mL) of biotin-labeled CD6 (PD1, PD2, and PD3), deleted in malignant brain tumors-1 (DMBT-1)/SAG- (pbs1), and CD5- (PD1) derived peptides (top panel), or rshCD5 and recombinant soluble human CD6 ectodomain (rshCD6) proteins (bottom panel) were added to 96-well ELISA plates sensitized with lipopolysaccharide (LPS) **(A)** or lipoteichoic acid (LTA) **(B)**. Following overnight incubation at 4°C bound peptides or proteins were developed by addition of horseradish peroxidase–streptavidin and 3,3’,5,5’-tetramethylbenzidine substrate and further readings at OD 405–620 nm. Results are expressed as mean ± SD of duplicates from one representative experiment of three performed. Statistical analysis was performed by two-way ANOVA (*P < 0.05; **P < 0.01; and ***P < 0.001). **(C)** Apparent *K*_d_ values and Hill coefficients for the binding of peptides and proteins in study to LPS and LTA determined by tryptophan fluorescence. Peptides and proteins (10 µg/mL) were titrated with or without increasing concentrations of LPS or LTA in phosphate buffered saline (PBS). Results are mean ± SD of three experiments. **(D)** Dynamic light scattering analysis of the hydrodynamic diameter of CD6 (PD1, PD2, and PD3) and DMBT-1/SAG (pbs1)-derived peptides (10 µg/mL) in PBS. The *y* axis represents the relative intensity of the scattered light; the *x* axis denotes the hydrodynamic diameter of the particles present in the solution. One representative experiment of four is shown.

Absence of bacterial agglutination does not fully exclude direct binding to bacterial PAMPs, so binding of biotin-labeled CD6-derived peptides to solid-phase bound LPS or LTA was tested by ELISA. As shown by Figures 3A,B, all CD6-derived peptides showed dose-dependent binding to LPS and LTA, similar to the pbs1 peptide and rshCD6 protein used as positive controls. As expected, no significant binding was observed for the CD5.PD1 peptide and the rshCD5 protein. These results confirm that CD6-derived peptides retain binding properties to LPS and LTA as reported for native CD6 but not CD5 proteins (14, 23).

To further characterize the interaction of CD6-derived peptides with LPS and LTA, the corresponding dissociation constants (*K*_d_) were determined by tryptophan fluorescence emission. As summarized in Figure 3C (Figure S1 in Supplementary Material), all CD6-derived peptides displayed high affinities for both LPS and LTA, being higher for CD6.PD1 and/or CD6.PD2 compared to CD6.PD3 (PD1 ≥ PD2 > PD3). *K*_d_ values for CD6.PD1 and CD6.PD2, but not CD6.PD3, are lower than for the prototypical DMBT-1/SAG.pbs1 peptide or the rshCD6 protein itself. The greater affinity of CD6.PD1 and CD6.PD2 for LPS, and LTA is not correlated with their bacterial agglutination properties, which are absent in CD6.PD2.

To determine whether self-aggregation properties of CD6-derived peptides are related to their agglutination properties, the hydrodynamic size of CD6-derived peptides in solution was analyzed by DLS. Results indicate that CD6-derived peptides formed particles of different hydrodynamic sizes according to their self-aggregation properties (Figure 3D). CD6.PD3 particles exhibited two peaks, at 630 ± 3 and 5.151 ± 7 nm, indicative of self-aggregation. In contrast, CD6.PD2 showed a single peak centered at 379 ± 5 nm, whereas CD6.PD1 showed two peaks at 321 ± 5 and 1.484 ± 8 nm, in line with the greater bacterial agglutination properties of CD6.PD3 (and CD6.PD1) with respect to CD6.PD2. Higher CD6.PD3 binding to immobilized LPS or LTA (Figures 3A,B) may respond to the fact that self-association of biotinylated peptide would produce an enhancement of the chromogenic signal. Whatever the case, the binding results univocally support the direct and substantial interaction of CD6-derived peptides with essential cell wall components from Gram-negative and Gram-positive bacterial strains.

**Figure 4:**
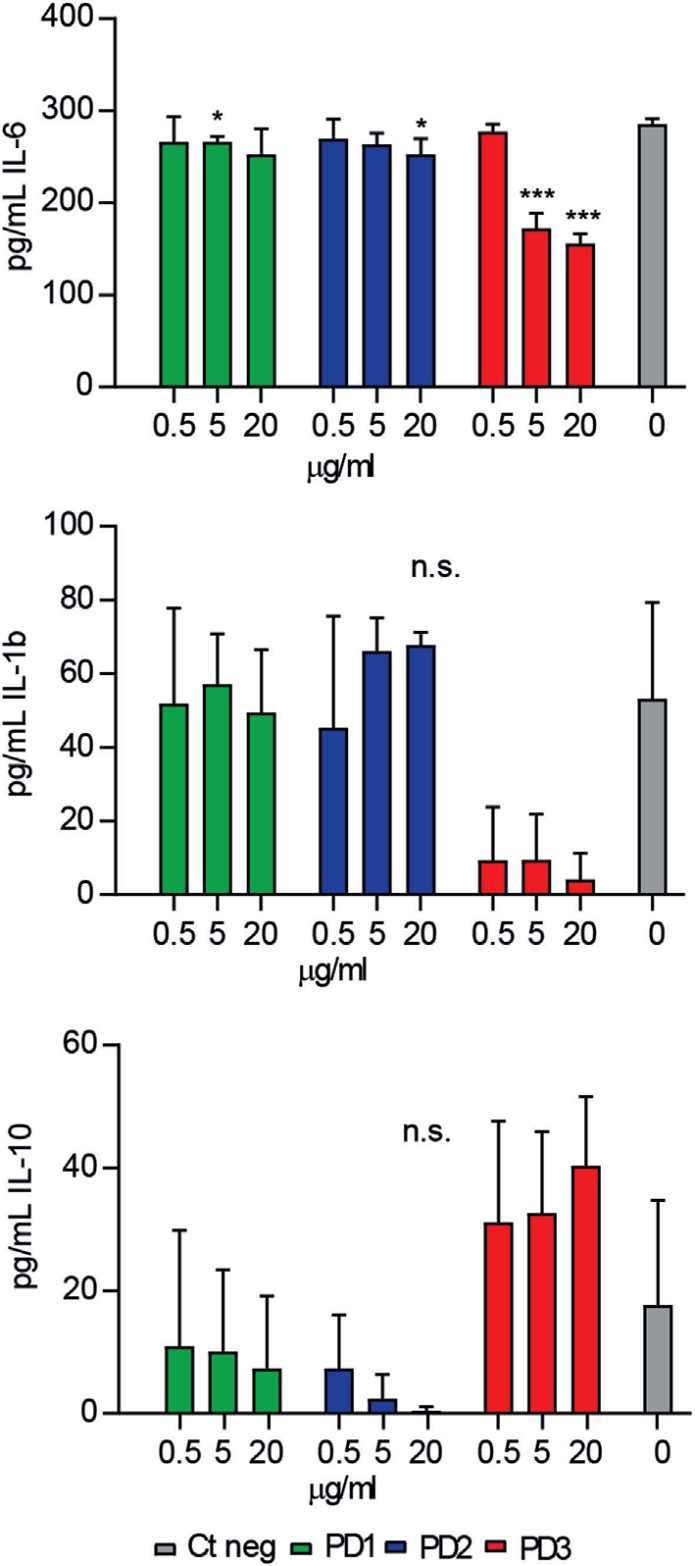
Effect of CD6-derived peptides on bacterial lipopolysaccharide (LPS)-induced cytokine release *in vitro* by mouse splenocytes. Total spleen cell suspension (2 × 10^5^) from C57BL/6 mice (*n* = 7) was stimulated in triplicate for 48 h with LPS (0.5 µg/mL), in the presence or absence of increasing concentrations (0.5, 5, and 20 µg/mL) of CD6-derived peptides (PD1, PD2, and PD3). Cytokine levels in culture supernatants were determined by ELISA and results expressed in pg/mL as mean ± SD of triplicates. Viability was >75% at 48 h in all experimental conditions. Statistical analysis was performed using a two-tailed Mann–Whitney test, with confidence intervals of 95% (n.s., not significant; *P < 0.05; **P < 0.01; and ***P < 0.001).

Next, the functional relevance of CD6-derived peptides interaction with key pathogenic bacterial products was explored *ex vivo*. To this end, the modulatory effects of increasing concentrations of CD6-peptides on cytokine release by mouse splenocytes exposed to LPS were tested. As illustrated by Figure 4, only CD6.PD3 showed significant dose-dependent inhibitory effects on pro-inflammatory IL-6 and IL-1β cytokine release, which reached statistical significance in the former case. The same CD6.PD3 peptide also induced a non-statistically significant increased release of the anti-inflammatory cytokine IL-10, as reported for rshCD6 (23). No significant effect was observed regarding TNF-α release for any of the CD6-derived peptides tested (data not shown).

### *In Vivo* Efficacy of CD6-Derived Peptides in CLP-Induced Septic Shock

**Figure 5:**
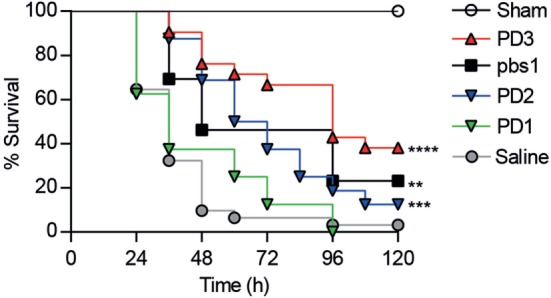
Comparative therapeutic effects of intravenous (*i.v*.) infused CD6-derived peptides on mouse survival following cecal ligation and puncture (CLP)-induced sepsis. C57BL/6J mice were *i.v*. infused with saline (*n* = 31) or single 6 mg/kg doses of unlabeled CD6 (PD1, *n* = 8; PD2, *n* = 16; PD3, *n* = 22) or DMBT-1/SAG (pbs1, *n* = 13) derived peptides 1 h post CLP induction. A sham group (*n* = 3) was included, which received saline. The average percent survival was analyzed over time for each group and compared to the saline-treated group using the long-rank *t*-test (**P < 0.01; ***P < 0.001, ****P < 0.0001).

The effects of CD6-derived peptides *in vivo* were tested in mice undergoing CLP-induced septic shock (27). To this end, a single intravenous (*i.v*.) dose (6 mg/kg) of the different CD6-derived peptides was infused to C57BL/6 mice 1 h post CLP-induction, and survival monitored thereafter. As shown in Figure 5, increased survival was observed among mice infused with CD6.PD2 (12.5%, *P* = 0.0005) and CD6.PD3 (36.36%, *P* < 0.0001) compared to the saline-treated group, a fact also observed in mice infused with the DMBT-1/SAG.pbs1 (23.07%, *P* = 0.0025). In contrast, no effects were evidenced for CD6.PD1.

**Figure 6:**
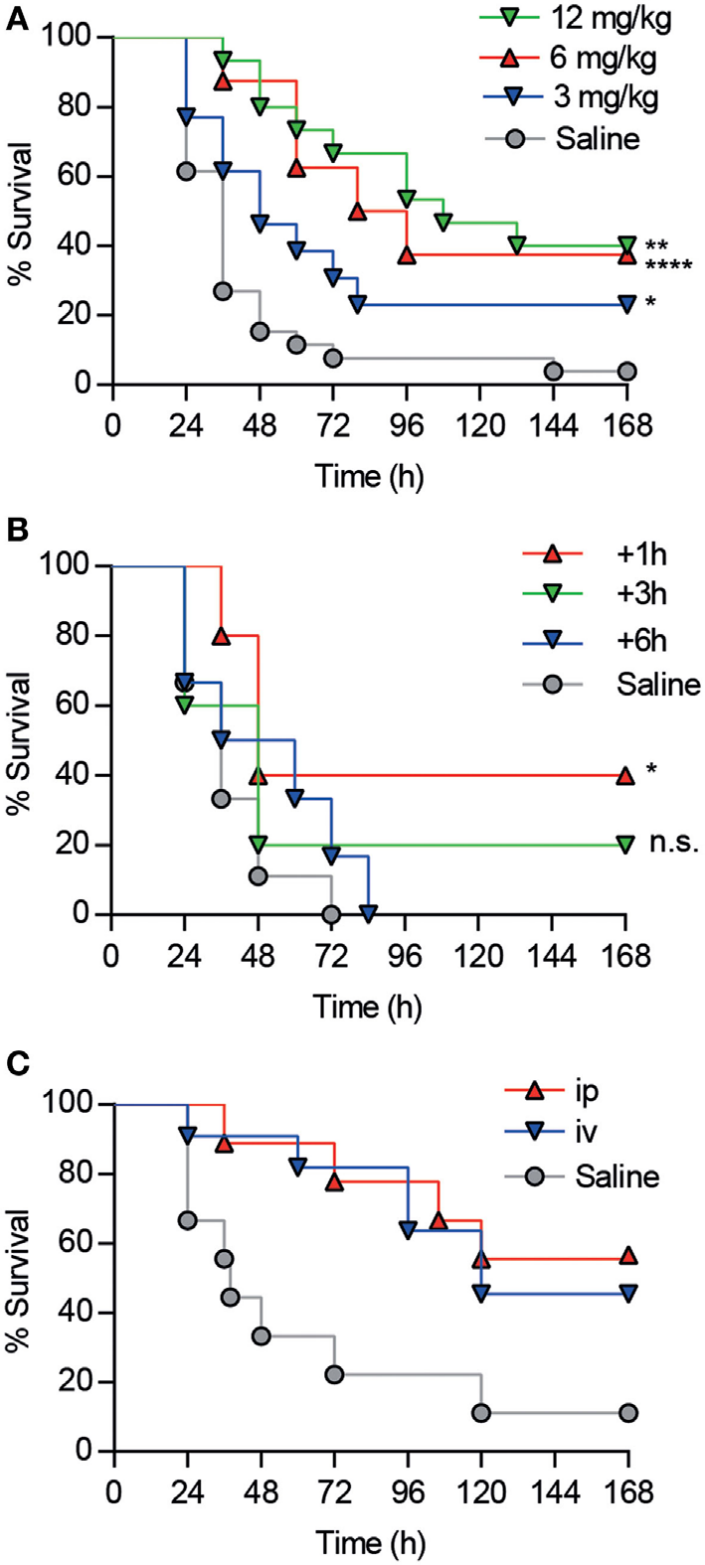
Analysis of dose-, time-, and *via*-dependent effects of CD6.PD3 infusion on mouse survival following cecal ligation and puncture (CLP)-induced sepsis. **(A)** C57BL/6 mice were intravenous (*i.v*.) infused 1 h post CLP with saline (*n* = 26) or single increasing doses (3 mg/kg, *n* = 13; 6 mg/kg, *n* = 15; and 12 mg/kg, *n* = 8) of unlabeled CD6.PD3 peptide. **(B)** C57BL/6 mice were *i.v*. infused with saline (*n* = 5) or 6 mg/kg CD6.PD3 at different times post CLP (+1 h, *n* = 5; +3 h, *n* = 5; and +6 h, *n* = 6). **(C)** C57BL/6J mice were *i.v*. (*n* = 11) or intraperitoneally (*n* = 9) infused with 6 mg/kg CD6.PD3 peptide or saline (*n* = 9) 1 h post CLP. In all cases, average percentage of survival was analyzed over time and compared with the saline-treated group using a log-rank *t*-test (n.s., not significant; *P < 0.05. **P < 0.01; and ***P < 0.001).

Since the *in vivo* protective properties of CD6.PD3 against septic shock excelled CD6.PD1 and CD6.PD2, additional experiments exploring its time-, dose-, and systemic via-dependent effects were performed. As shown in Figures 6A–C, maximal survival rates post CLP were obtained following CD6.PD3 infusion at 6 or 12 mg/kg doses (37.5 and 40%, respectively, vs 23.08% at 3 mg/kg), and 1 h after CLP induction (40% at + 1 h vs 20% at + 3 h), irrespective of the *i.v*. or intraperitoneal (*i.p*.) infusion pathway used.

Next, the effect of the CD6.PD3 peptide on serum cytokine levels and bacterial load post CLP were further monitored. To this end, C57BL/6 mice undergoing CLP-induced septic shock were treated with saline or CD6.PD3 peptide under the previously stated optimal conditions (single *i.v*. infusion of 6 mg/kg at 1 h post CLP) and thereafter bled and sacrificed at 4 and 20 h later, respectively.

**Figure 7:**
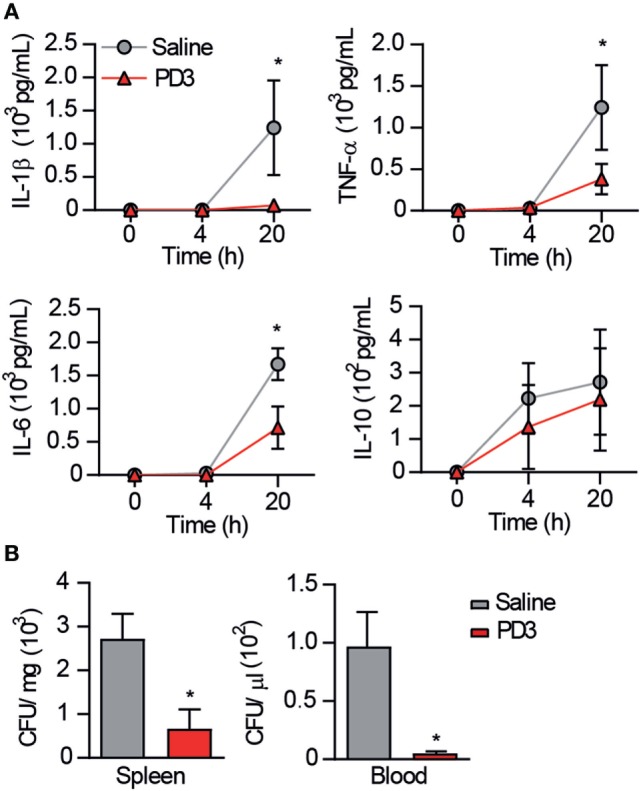
Effect of CD6.PD3 therapeutic infusion on cytokine plasma levels and bacterial load following cecal ligation and puncture (CLP)-induced sepsis. **(A)** C57BL/6J mice were intravenous infused 1 h post CLP with saline (*n* = 7) or CD6.PD3 peptide (6 mg/kg; *n* = 9), and cytokine plasma levels were monitored by ELISA at different time points (4 and 20 h) thereafter. Data are expressed as mean ± SD. **(B)** Same mouse groups as in **(A)** were monitored for blood and spleen bacterial load at different time points (4 and/or 20 h) following CLP induction. Data are expressed as mean ± SD of colony-forming units (CFU)/mg (spleen) or CFU/μL (blood). In all cases, statistical differences were evaluated using a two-tailed Student *t*-test (*P < 0.05).

**Figure 8:**
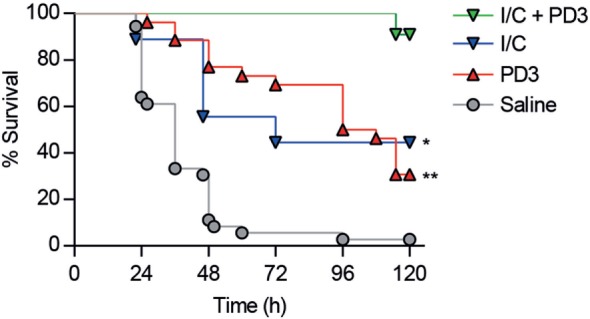
Additive effects of combined administration of CD6.PD3 and Imipenem/Cilastatin on mouse survival following cecal ligation and puncture (CLP)-induced sepsis. C57BL/6J mice were therapeutically infused 1 h post CLP with saline (*n* = 31), CD6.PD3 (6 mg/kg intravenous; *n* = 25), Imipenem/Cilastatin (I/C, 50 mg/kg/12h intraperitoneal; *n* = 9), or a combination of the two later (*n* = 11). The average percent survival was analyzed over time for each group and compared to the I/C plus CD6.PD3 group using the long-rank *t*-test (*P < 0.05 and **P < 0.01).

As shown in Figure 7A, CD6.PD3-treated mice exhibited lower levels (*P* < 0.05) of the pro-inflammatory cytokines IL-1β, IL-6, and TNF-α at 20 h post CLP, compared to the saline-treated group. Similarly, the same CD6.PD3-treated mice also produced lower CFU isolated from blood and spleen when sacrificed at 20 h post CLP, compared to the control group (Figure 7B). These results indicate that the CD6.PD3 peptide retains the therapeutic properties reported for the rshCD6 protein in experimental models of septic shock (24). This also holds for septic mice simultaneously treated with CD6.PD3 and the broad-spectrum bactericidal antibiotic Imipenem/Cilastatin. As illustrated in Figure 8, the combined administration of CD6.PD3 (6 mg/kg *i.v*.) and Imipenem/Cilastatin (50 mg/kg/12 h *i.p*.) 1 h post CLP-induced septic shock resulted in additive/synergistic effects on mice survival (90.9%), compared to either treatment individually (30.8 and 44.4%, respectively).

## Discussion

Membrane-bound and soluble forms of the lymphocyte receptor CD6 act as receptors for bacterial PAMPs of Gram-negative or Gram-positive origin (14, 23). The bacterial-binding properties of host receptors can be exploited as a source of alternative/adjunctive therapies for the prevention and/or treatment of severe infectious processes with little or no response to conventional antibiotic therapy (3), as is the case of the CD6 receptor in proof of principle studies performed by our group. The prophylactic or therapeutic infusion of a single dose of rshCD6 increases survival of mice undergoing lethal septic shock following mono- or polymicrobial peritonitis (23, 24), concomitant with lower levels of circulating pro-inflammatory cytokines and lower bacterial loads. Importantly, rshCD6 infusion shows additive effects when combined with a bactericidal antibiotic (Imipenem/Cilastatin) and displays efficacy against drug-resistant Gram-negative and Gram-positive strains (Colistin-resistant *A. baumannii* and MRSA) (23, 24). The latter results from CD6 specifically targeting LPS and LTA/PGN, which are key pathogenic bacterial structures not easily amenable to antibiotic-induced mutation, as this would impact bacterial viability and/or pathogenicity.

In the present work, we have identified short (11-mer long) CD6-derived intradomain peptides retaining *in vitro* and *in vivo* bacterial-recognition properties of the native CD6 protein. Such sequences (CD6.PD1, CD6.PD2, and CD6.PD3) map at surface-accessible sites of the three SRCR domains of CD6 and are homologous to the 11-mer consensus peptide (pbs1) identified in DMBT-1/SAG (10). While (i) similar homologous sequences from some SRCR-SF members possessing the minimal VEVLxxxxW motif do not bind to bacteria (10), and (ii) none of the CD6-derived peptides fully matched the consensus motif, all the three CD6-derived peptides interact with both LPS and LTA albeit with different *K*_d_, and with varied *in vitro* and *in vivo* functional properties (e.g., bacterial agglutination or prevention of CLP-induced mortality). This is best illustrated by CD6.PD3, which excelled in *in vitro* functional assays, while exhibited lowest affinity for LPS and LTA. A plausible explanation arises from the hydrodynamic diameter of CD6-derived peptides in solution, in which CD6.PD3 shows higher self-aggregation values. A number of antimicrobial peptides (including SP-B^N^, cathelicidin LL-37, dermaseptin S9, and temporins B and L) have been reported to form aggregates (30, 33–35), suggesting a mechanistic connection between peptide aggregation and antimicrobial activity.

The CD6.PD3 peptide also provided better *in vivo* results when assayed for therapeutical purposes in the mouse model CLP-induced septic shock compared with the other CD6-derived peptides (*P* = 0.0405 for CD6.PD2 and *P* < 0.0005 for CD6.PD1) but not with the prototypical DMBT-1/SAG.pbs1 peptide (*P* > 0.194). It should be noted in this regard that neither the DMBT-1/SAG.pbs1 nor the CD163p2 peptides have been tested before for their anti-bacterial efficacy in *in vivo* mouse models of infection. Another remarkable finding is CD6.PD3 peptide’s additive/synergistic effect on mice survival when coadministered with Imipenem/Cilastatin, a member of the carbapenem family considered as first-choice treatment in critical care patients undergoing sepsis (36). Therefore, CD6.PD3 gathers most of the anti-bacterial properties of rshCD6, thus constituting a good cost-effective alternative to the latter, as well as a good adjunctive strategy to antibiotic therapy.

CD6.PD1 and CD6.PD2 high affinity (and also CD6.PD3) to LPS and LTA makes these peptides suitable candidates for new supportive non-antibiotic strategies against sepsis. One such possibility would be the adsorption of circulating bacterial toxins by CD6-derived peptides covalently coupled to a solid phase. Preliminary results obtained by incubating an endotoxin solution (50 UI/mL LPS) with Eupergit^®^ beads coated with different CD6-derived peptides (CD6.PD2 and CD6.PD3) or proteins (HSA, rshCD5, and rshCD6) for different periods of time support this approach (Figure S2 in Supplementary Material). CD6.PD2-, CD6.PD3-, and rshCD6-coated beads reduced endotoxin levels (as detected by LAL assays) compared to HSA- and rshCD5-coated controls. The use of CD6-derived peptides for extracorporeal hemoperfusion would deserve further exploration since it would have advantages over existing devices such as Polymyxin B-immobilized fiber blood-purification columns (37): (i) the reported affinity of the LPS/Polymixin B interaction (*K*_d_ 100–900 nM, depending on the Gram-negative strain used) (38) is lower than that of CD6.PD1 and CD6.PD2 (*K*_d_ 3.5 ± 0.3 and 35 ± 2 nM, respectively), and (ii) Polymixin B mainly binds to LPS, while CD6.PD1 and CD6.PD2 also bind LTA with affinities of *K*_d_ 0.39 ± 0.06 and 0.31 ± 0.04 nM, respectively. The latter would support the use of those CD6-derived peptides in the case of Gram-positive infections, responsible for over 50% of sepsis (39).

In conclusion, the present findings that short (11-mer) peptide sequences can retain the bacterial-binding properties of the whole extracellular region of CD6 open cost-effective opportunities for developing new adjunctive alternatives to currently available sepsis treatment. The complex physiology of the sepsis response requires multi-disciplinary and simultaneously study of the various time-dependent factors determining short- and long-term sepsis outcome.

## Ethics Statement

The protocol was approved by the Animal Experimentation Ethical Committee of the University of Barcelona.

## Author Contributions

MM-F, CCasals, and FL designed the experiments; MM-F, CCatalà, MV-DA, OC, VF-A, NA-B, SC-L, MC-F, CCasals, and FL performed the experiments and/or analyzed data; and MM-F, MC-F, CCasals, and FL wrote the manuscript.

## Conflict of Interest Statement

The authors declare that the research was conducted in the absence of any commercial or financial relationships that could be construed as a potential conflict of interest.
