# Supplementary material for: Conserved Bacterial-Binding Peptides of the Scavenger-Like Human Lymphocyte Receptor CD6 Protect From Mouse Experimental Sepsis
Source: Front Immunol. 2018 Apr 12;9:627. doi: 10.3389/fimmu.2018.00627 (PMC5906529; doi:10.3389/fimmu.2018.00627)

Supplementary Material

Conserved Bacterial-binding Peptides of the Scavenger-like Lymphocyte Receptor CD6 Protect from Experimental Sepsis

Mario Martínez-Florensa[1], Cristina Català[1], María Velasco-de Andrés[1], Olga Cañadas[2,3], Víctor Fraile-Ágreda[2,3], Sergi Casadó-Llombart[1], Noelia Armiger-Borràs[1], Marta Consuegra-Fernández[1], Cristina Casals[2,3], Francisco Lozano[1,4,5] *

[1]Immunoreceptors of the innate and adaptive system, Institut d'Investigacions Biomèdiques August Pi i Sunyer (IDIBAPS), Barcelona (Spain). [2]Centro de Investigación Biomédica en Red de Enfermedades Respiratorias (CIBERES), Instituto de Salud Carlos III, Madrid (Spain). [3]Departmento de Bioquímica y Biología Molecular I, Universidad Complutense de Madrid, Madrid (Spain). [4]Servei d'Immunologia, Centre de Diagnòstic Biomèdic, Hospital Clínic de Barcelona, Barcelona (Spain). [5]Departament de Biomedicina, Facultat de Medicina, Universitat de Barcelona, Barcelona (Spain)

Running title: *Bacterial-binding CD6 peptides and sepsis*

(*)Correspondence:
Francisco Lozano
E-mail: flozano@clinic.ub.es

1 Supplementary Data

Protein/peptide immobilization to Eupergit® beads

Proteins or peptides (2.5 mg each) were immobilized via NH_2 groups on macroporous acrylic EUPERGIT® beads (0.2 g; Röhm-Pharma GmbH, Germany) following a previously described protocol [1].

Endotoxin assay

LPS detection was performed by using the turbidimetric-kinetic Limulus Amebocyte Lysate (LAL) kit- QCL (50-650U, Lonza) following manufacturer's instructions. Protein/peptide-coated EUPERGIT® beads were incubated 1:1 (v:v) with the provided endotoxin solution (50 UI/mL) for different periods of time (0-150 min) at room temperature following manufacturer's instructions.

References

1. Zimmermann, M., Busch, K., Kuhn, S., Zeppezauer, M. (1999) Endotoxin adsorbent based on immobilized human serum albumin. *Clin Chem Lab Med.* **37**, 373–379.

2 Supplementary Figures and Tables

2.1 Supplementary Figures

Supplementary Figure S1. **Binding of rshCD6 protein, and CD6 (CD6.PD1, CD6.PD2, and CD6.PD3) and DMBT-1/SAG (pbs1)-derived peptides to LPS and LTA determined by tryptophan fluorescence.** Peptides (10 µg/mL) were titrated with or without increasing concentrations of Re-LPS (upper panel) or LTA (lower panel) in PBS. Peptide samples (with and without either Re-LPS or LTA) and blank samples (Re-LPS or LTA alone) were excited at 295 nm, and the emission spectra recorded from 300 to 400 nm. Results are expressed as the change in peptide fluorescence (ΔF) at the wavelength of the emission maxima (353 nm for the peptides and 337 nm for rshCD6 protein) in the presence and absence of either Re-LPS or LTA. Results are means ± SD of 3 experiments. Peptide fluorescence changes at 353 nm were fitted to the Hill equation. The apparent K_d values and Hill coefficients for the binding of the different peptides to LPS and LTA are shown.

Supplementary Figure S2. Endotoxin adsorption assays on immobilized CD6-derived peptides and proteins. Eupergit® beads coated with different proteins (HSA, rshCD5, and rshCD6) or CD6-derived peptides (PD2, PD3) were incubated for different periods of time (0, 30, 90 and 150 min) with a 50 UI/mL endotoxin solution. LAL activity of LPS in supernatants was then monitored along time and the OD 405-620 nm represented. Shown are triplicates of a representative experiment from two independent performed. Statistical analysis was done by using the 2-tailed paired T test with 95% confidence interval (*, P <0.05; **, P <0.01; ***, P <0.001).

Figure S1

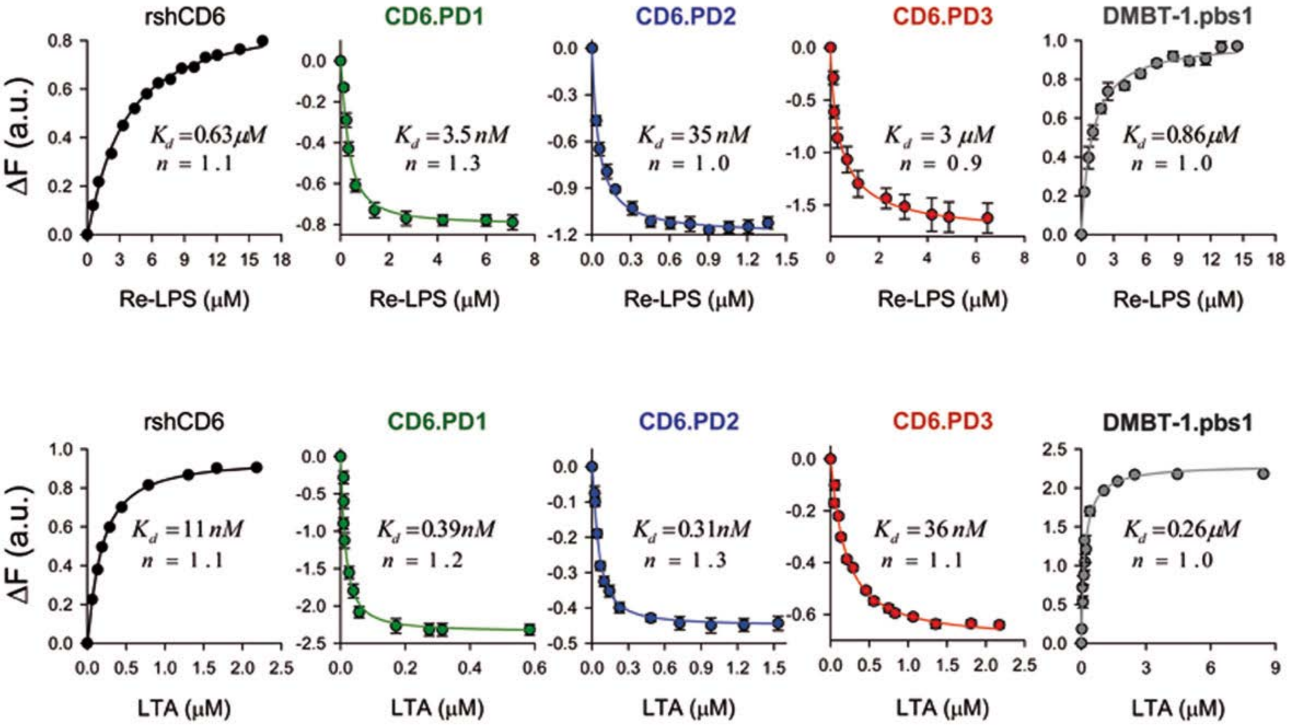

Figure S2

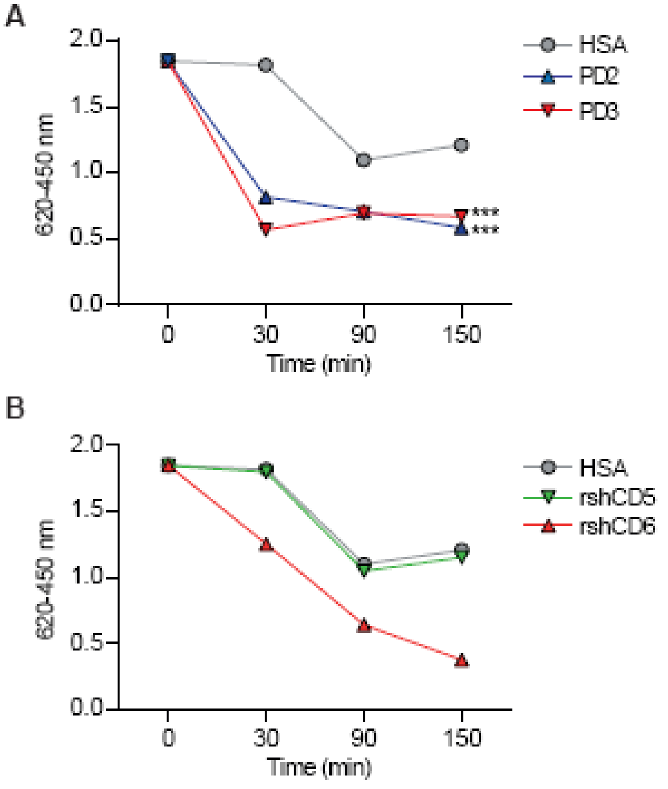

Supplement: Supplementary file 1 [file Presentation_1.PDF]
